# Shank3B deficiency disrupts GABAergic synaptic transmission in pyramidal neurons of the medial prefrontal cortex region in autism spectrum disorder

**DOI:** 10.1186/s13041-026-01289-z

**Published:** 2026-03-09

**Authors:** Heqing Yin, Ke Sun, Chenyang Wang, Songqiao Fan, Jiaqi Wang, Shitai He, Hui meng Lei, Shuo Pang, Jun Chen, Guojun Zhang

**Affiliations:** 1 The Laboratory of Neurological Disorders and Brain Cognition, National Center for Children’s Health, Beijing Pediatric Research Institute, Beijing Children’s Hospital, Capital Medical University, Beijing, 100045 China; 2 Laboratory for Clinical Medicine, Capital Medical University, Beijing, 100045 China; 3 Key Laboratory of Major Diseases in Children, Ministry of Education, Beijing, 100045 China; 4 Laboratory for Clinical Medicine, Beijing Key Laboratory of Neural Regeneration and Repair, Key Laboratory for Neurodegenerative Diseases of the Ministry of Education, Department of Neurobiology, School of Basic Medical Sciences, Capital Medical University, Beijing, 100069 China

**Keywords:** Autism spectrum disorder, *Shank3b* knockout mice, Medial prefrontal cortex, Synapse, GABAergic synaptic transmission

## Abstract

Mutations in the *SHANK3B* gene have been strongly implicated in the pathogenesis of autism spectrum disorder (ASD). The medial prefrontal cortex (mPFC) is integral to emotional processing and social behavior, and its atypical development is closely associated with ASD pathogenesis. However, the electrophysiological characteristics of pyramidal neurons within the mPFC and the mechanisms of their synaptic transmission remain inadequately characterized. In the present study, we conducted whole-cell patch-clamp recordings on mPFC pyramidal neurons in *Shank3b* knockout mice. We observed significant alterations in the membrane properties and excitability of mPFC pyramidal neurons in *Shank3b* knockout mice; these were accompanied by reduced inhibitory postsynaptic currents and deficiencies in γ-aminobutyric acid (GABA) release or GABA_A_ receptor transport. Additionally, RNA sequencing analysis of PFC tissue revealed differentially expressed genes in *Shank3b* knockout mice compared with WT mice, with these genes enriched in synaptic function and calcium channel signaling pathways. These findings are consistent with our ultrastructural observations of a reduced postsynaptic density at excitatory synapses, which may further contribute to the impaired number and morphology of pyramidal neurons in the mPFC. Our research offers new insights into the disruption of PFC circuitry that is caused by *Shank3b* deficiency and establishes connections between the pathophysiological mechanisms underlying ASD and synaptic structural anomalies, ion channel dysregulation, and excitatory/inhibitory imbalances. Together, these findings highlight the importance of *Shank3b*-mediated regulation of GABA signaling and modulation of intrinsic excitability as prospective therapeutic targets for ASD.

**Supplementary Information:**

The online version contains supplementary material available at 10.1186/s13041-026-01289-z.

## Introduction

Autism spectrum disorder (ASD) is a neurodevelopmental condition that is characterized by core symptoms such as deficits in communication, impaired social interaction, and repetitive or restricted interests and behaviors [1]. In recent years, the prevalence of ASD has been gradually increasing, and ASD has emerged as an important public health concern worldwide. The World Health Organization estimates that the global prevalence of ASD is approximately 1%–2% [2, 3]. The etiology of ASD is complex and involves the interplay of multiple factors such as genetics and neurobiology. Ongoing research has identified several risk gene variants, including those in SH3 and multiple ankyrin repeat domains 3 (*SHANK3*), neurexin (*NRXN*), and discs large MAGUK scaffold protein 4 (*PSD95* or *DLG4*), which all significantly influence susceptibility to ASD [4–8]. Many of these genes are associated with synaptic function, the excitatory/inhibitory (E/I) balance, and brain connectivity [4]. Consequently, disruptions in the E/I balance and synaptic function may serve as critical pathophysiological drivers of ASD.

Among the high-risk genes associated with ASD, *SHANK3* is notable for its critical role in synaptic scaffolding and glutamate receptor trafficking [9]. *SHANK3* encodes a key postsynaptic density (PSD) protein at glutamatergic synapses; its mutations disrupt PSD integrity and dendritic spine dynamics, leading to impaired synaptic plasticity and neuronal network dysfunction [9, 10]. Notably, *Shank3*-deficient mice exhibit ASD-like behaviors including social interaction deficits and repetitive grooming, thereby making them a robust model for studying synaptic pathophysiology in ASD [5, 11].

The E/I balance is fundamental to the maintenance of neural circuit homeostasis, and its disruption is posited as a central mechanism underlying ASD [12]. Supporting this hypothesis, both postmortem analyses and animal model studies have revealed altered levels of proteins associated with glutamate and γ-aminobutyric acid (GABA) in the brain tissues of individuals with ASD or animal models of ASD [13, 14]. Variations in the E/I balance are evident across different brain regions in *Shank3* mouse models of ASD. Specifically, region-specific abnormalities have been identified in the hippocampal and cortical neural circuits of a *Shank3* mutation model. In this context, the deletion of *Shank3* disrupts PSD-95/glutamate receptor 1 interactions and compromises α-amino-3-hydroxy-5-methyl-4-isoxazolepropionic acid (AMPA) receptor stability, whereas the dysregulation of potassium/sodium hyperpolarization-activated cyclic nucleotide-gated channel 1 channels may hinder dendritic integration [9, 15]. Additionally, synaptic plasticity mechanisms, such as long-term potentiation and long-term depression, are notably sensitive to *Shank3* dosage, with research indicating the existence of region-specific impairments in hippocampal and cortical neural circuits [9]. Consequently, investigating the deficits in E/I synaptic plasticity and neurophysiology across various brain regions in a *Shank3b* knockout (*Shank3b*^−/−^) ASD mouse model may offer crucial experimental evidence for understanding the neural basis of this disorder.

The prefrontal cortex (PFC) is a cortical region that is located at the anterior part of the brain. It serves as a crucial center for the internal coordination of sensory and motor processes at a fundamental level, thereby enabling the brain to adapt to internal objectives and respond accordingly [16, 17]. In addition to its involvement in emotional and social behaviors, the PFC provides “top-down” executive control, particularly when behaviors are directed by internal states or goals [17]. The medial PFC (mPFC), which is a core subregion of the PFC, is particularly focused on emotion regulation and social cognition [18]. Upward projections from the mid-dorsal thalamus to the anterior cingulate cortex have been implicated in social interactions [19]. Recent research indicates that the mPFC exhibits dysfunction in the *Shank3b*^−/−^ mice; this is characterized by structural and functional alterations in its pyramidal neurons (PNs), such as reduced dendritic branching and dysregulated N-methyl-D-aspartic acid (NMDA)/AMPA receptor ratios [9, 20]. Nonetheless, the precise mechanisms linking *Shank3* deficiency to the E/I balance and synaptic function within the mPFC remain inadequately understood.

In the present study, we used *Shank3b*^−/−^ mice as a model to explore neuromodulatory mechanisms within the mPFC. Using electrophysiological membrane clamp techniques, we assessed the intrinsic physiological properties and neuronal excitability of neurons in the mPFC of *Shank3b*^−/−^ mice. Our findings indicate that *Shank3b* deficiency leads to increased intrinsic neuronal excitability and alterations in the frequency and amplitude of miniature inhibitory postsynaptic currents (mIPSCs). Furthermore, we observed significant morphological impairments in mPFC PNs and synapses in *Shank3b*^−/−^ mice, suggesting that these structural abnormalities may contribute to the observed deficits in neuronal excitability and plasticity. RNA sequencing (RNA-seq) analysis also corroborated the presence of significant alterations in synapse-associated signaling pathways in the PFC of *Shank3b*^−/−^ mice.

## Materials and methods

### Animals

In this study, we used male and female *Shank3b*^−/−^ mice, aged 6–8 weeks, alongside their wild-type (WT) counterparts. The mice were maintained under controlled temperature and humidity conditions, with a 12-h light/dark cycle, and were provided with unrestricted access to food and water. All experimental protocols received approval from the Institutional Animal Care and Use Committee of Capital Medical University (Approval no. AEEI-2024-109). Genotypes were confirmed using polymerase chain reaction analysis, as previously documented.

#### Open field test

An open field test arena with dimensions of 50 × 50 × 40 cm^3^ was used. At the start of the test, mice were placed in the center, and their free exploration was recorded for 10 min. The time and distance traveled in the central area, as well as the total distance traveled, were analyzed using SMART v.3.0 software.

#### Self-grooming test

To measure self-grooming, mice were individually placed into a standard mouse cage. After a 5-min habituation period, mice were video recorded for 10 min using a horizontally mounted camera. The total time spent self-grooming was scored by a trained observer who was blind to the treatment group.

#### Three-chamber social test

Each test mouse was first placed in the middle chamber and allowed to explore for 5 min. After the habituation period, an unfamiliar C57BL/6 J male (stranger 1) that had had no prior contact with the subject mouse was placed in one of the side chambers, and a novel object was placed in the opposite side. Both doors to the side chambers were then unblocked and the subject was allowed to explore the entire social test box for 10 min. The amount of time spent in each chamber was measured. At the end of the first 10-min session, each mouse was then tested in a second 10-min session to quantify social preference for a second stranger. In this part of the test, a second, unfamiliar mouse was placed in the chamber in which the object had been placed during the first 10-min session. The test mouse then had a choice between the first, already-investigated unfamiliar mouse (stranger 1) and the novel, unfamiliar mouse (stranger 2). The amount of time spent in each chamber of the apparatus during the second 10-min session was measured.

### Drugs

Tetrodotoxin (TTX), 6-cyano-7-nitroquinoxaline-2,3-dione, D-(-)-2-amino-5-phosphonopentanoic acid, and bicuculline were procured from Tocris Bioscience and administered via bath application at concentrations of 1 μM, 10 μM, 50 μM, and an unspecified concentration, respectively.

### Electrophysiological recordings in mPFC brain slices

Anesthesia was induced using isoflurane prior to decapitation. The mouse brains were swiftly extracted and immersed in ice-cold cutting solution composed of 213 mM sucrose, 3 mM KCl, 1.3 mM NaH_2_PO_4_, 23 mM NaHCO_3_, 0.5 mM CaCl_2_, 5 mM MgCl_2_, and 10 mM glucose (pH 7.4, 300–310 mOsm). Coronal slices of the mPFC, each with a thickness of 300 μm, were prepared using a vibratome (DTK-1000N, DOSAKA) and subsequently transferred to a holding chamber containing artificial cerebrospinal fluid (125 mM NaCl, 2.5 mM KCl, 1.3 mM NaH_2_PO_4_, 25 mM NaHCO_3_, 2 mM CaCl_2_, 1.3 mM MgCl_2_, and 10 mM glucose; pH 7.4, 300–310 mOsm). This solution was saturated with a gas mixture of 95% O_2_ and 5% CO_2_ and maintained at a temperature of 32 °C for 1 h. Electrophysiological recordings were conducted at ambient room temperature (22–24 °C).

### Whole-cell patch-clamp recordings

To evaluate intrinsic membrane properties and synaptic currents, whole-cell recordings were obtained from layer V PNs in the mPFC under visual guidance using differential interference contrast microscopy. Borosilicate glass pipettes (3–5 MΩ resistance) were filled with intracellular solution containing: 124 mM K-gluconate, 6 mM KCl, 10 mM N-2-hydroxyethylpiperazine-N'-2-ethanesulfonic acid, 5 mM egtazic acid, 1 mM CaCl_2_, 0.5 mM MgCl_2_, 5 mM Mg-ATP, 0.5 mM Na-GTP, and 12 mM phosphocreatine (pH 7.3, 290–300 mOsm). Neurons were voltage-clamped at − 70 mV (unless specified) using a HEKA EPC10, and data were acquired at 10 kHz (Patch Master). Series resistance was monitored and compensated by ≥ 70%, and cells were discarded if the series resistance was > 30 MΩ or unstable.

### Membrane intrinsic properties

Passive membrane properties (input resistance and membrane capacitance) and action potential (AP) parameters were assessed using hyperpolarizing and depolarizing current steps (0 to + 200 pA, 500 ms duration). AP amplitude was measured from threshold to peak; the threshold was defined as the membrane potential at which dV/dt exceeded 20 V/s, the half-width was the width at half-maximal amplitude, and afterhyperpolarization (AHP) was the minimum membrane potential post-AP. Current–voltage relationships were generated by plotting the steady-state membrane potential against the injected current.

### Spontaneous excitatory postsynaptic currents (sEPSCs)

sEPSCs were recorded from PNs using a K-gluconate-based pipette filling solution, following the completion of current-clamp recordings. The membrane potential was clamped at − 70 mV and sEPSCs were recorded for 5 min.

### Spontaneous IPSCs (sIPSCs)

sIPSCs were recorded using a cesium-based pipette filling solution (pH 7.2, 280 mOsm) containing: 120 mM Cs-methanesulfonate, 10 mM CsCl, 10 mM N-2-hydroxyethylpiperazine-N'-2-ethanesulfonic acid, 1 mM egtazic acid, 2 mM QX314, 0.1 mM spermine, 4 mM Mg-ATP, 0.3 mM Na-GTP, and 10 mM phosphocreatine. The membrane potential was clamped at 0 mV and sIPSCs were recorded for 5 min.

### Miniature EPSCs (mEPSCs) and IPSCs (mIPSCs)

mEPSCs and mIPSCs were recorded using the aforementioned pipette filling solution in the presence of TTX (1 µM) to block APs. For miniature events, data were analyzed using MiniAnalysis software (Synaptosoft) with an amplitude threshold of 5 pA.

### Hematoxylin and eosin and Nissl staining

Paraffin sections were sequentially immersed in Environmentally Friendly Dewaxing Transparent Liquid I for 20 min, Environmentally Friendly Dewaxing Transparent Liquid II for 20 min, anhydrous ethanol I for 5 min, anhydrous ethanol II for 5 min, and 75% ethyl alcohol for 5 min. They were then rinsed with tap water before undergoing staining.

Hematoxylin staining: Sections were immersed in hematoxylin solution for 3–5 min before being rinsed with tap water. Next, the sections were treated with hematoxylin differentiation solution, rinsed with tap water, treated with hematoxylin bluing solution, and rinsed with tap water. The sections were then placed in 95% ethanol for 1 min, eosin dye for 15 s, absolute ethanol I for 2 min, absolute ethanol II for 2 min, absolute ethanol III for 2 min, normal butanol I for 2 min, normal butanol II for 2 min, xylene I for 2 min, and xylene II for 2 min, before being sealed with neutral gum.

Nissl staining: The tissue sections were treated with dye solution for 2–5 min before being rinsed with tap water. For differentiation, 0.1% glacial acetic was used; the reaction was terminated using running water and the differentiation degree was controlled under a microscope. After being washed with tap water, the sections were dried in the oven.

### Transmission electron microscopy (TEM)

Mice were deeply anesthetized and transcardially perfused with heparinized normal saline, followed by 2.5% glutaraldehyde and 1% paraformaldehyde in 0.1 M phosphate buffer (PB; pH 7.4). The cortex was removed, and TEM analysis was performed as previously described [15]. Briefly, the cortex was removed from the whole brain, postfixed in the same fixative buffer for 2 h, and stored in PB overnight at 4 °C. The somatosensory barrel field cortex was cut transversely on a vibratome at 70 µm. The sections were then osmicated with 0.5% OsO_4_ in 0.1 M PB for 1 h, dehydrated in graded alcohols, flat-embedded in EMbed 812 (Electron Microscopy Sciences), and cured for 48 h at 60 °C. Ultrathin 60-nm sections were cut and mounted on Formvar-coated single-slot grids. Sections were stained with uranyl acetate and lead citrate before being examined with an electron microscope (JEM-1400plus). The width and thickness of PSD clefts were analyzed using ImageJ 1.48 (https://imagej.nih.gov/).

### RNA-seq analyses

Experimental mice in the WT and *Shank3b* groups were sacrificed at 8 weeks and the mPFC was harvested for RNA-seq, which was performed by Annoroad Gene Technology (Beijing) Co., Ltd. The purity, concentration, and integrity of the RNA extracted from the hippocampus were assessed, and sequencing libraries were generated based on the extracted RNA. The resulting sequencing libraries were clustered and processed, and differentially expressed genes (DEGs) between the WT and *Shank3b* groups were identified using the DEseq2 package in R. Genes with an absolute fold-change > 1 and adjusted *p*-value < 0.05 were considered DEGs. Next, the Gene Ontology (GO) and Kyoto Encyclopedia of Genes and Genomes (KEGG) pathways that were enriched for DEGs were analyzed using the cluster Profile package in R, to explore the functional annotation of DEGs with adjusted *p*-values < 0.05. We mapped the pathways using the cnetplot package in R to interpret pathway-to-pathway correlations. Protein–protein interaction (PPI) networks were constructed using the STRING network; the resulting list of DEGs was uploaded to the STRING online database (confidence score cutoff > 700) to obtain protein–protein interaction network data and was plotted using cytoscape software. RNA-seq data were deposited in the National Center for Biotechnology Information BioProject database (http://www.ncbi.nlm.nih.gov/bioproject) with accession number: PRJNA907930.

### Data analysis

Data were analyzed offline using Clampfit 10.7 (Molecular Devices), MiniAnalysis, and Igor Pro (WaveMetrics). Statistical comparisons were performed using Student’s t-test, the Mann–Whitney U test, or one-way analysis of variance followed by post hoc tests, as appropriate. Data are presented as the mean ± standard error of the mean, with *p* < 0.05 considered significant.

## Results

The *Shank3b*^*−/−*^ mouse has been well-characterized in terms of displaying ASD-related behavioral phenotypes [1, 2]. In our prior work, we validated these findings via behavioral experiments; *Shank3b*^*−/−*^ mice indeed exhibited ASD-like phenotypes in the open field test and three-chamber social interaction test. In the present study, *Shank3b*^*−/−*^ mice exhibited altered locomotor activity, increased anxiety-like behavior, and impaired social interaction (particularly in initiating social engagement), whereas their social novelty discrimination differed in the time allocated and the associated sniffing duration (Supplement 1. A–O).

Following the validation of our initial behavioral assays, we performed additional analyses using electrophysiological, histological, and molecular techniques, among others.

### Altered intrinsic excitability of mPFC PNs in ***Shank3b***^−/−^ mice

**Fig. 1:**
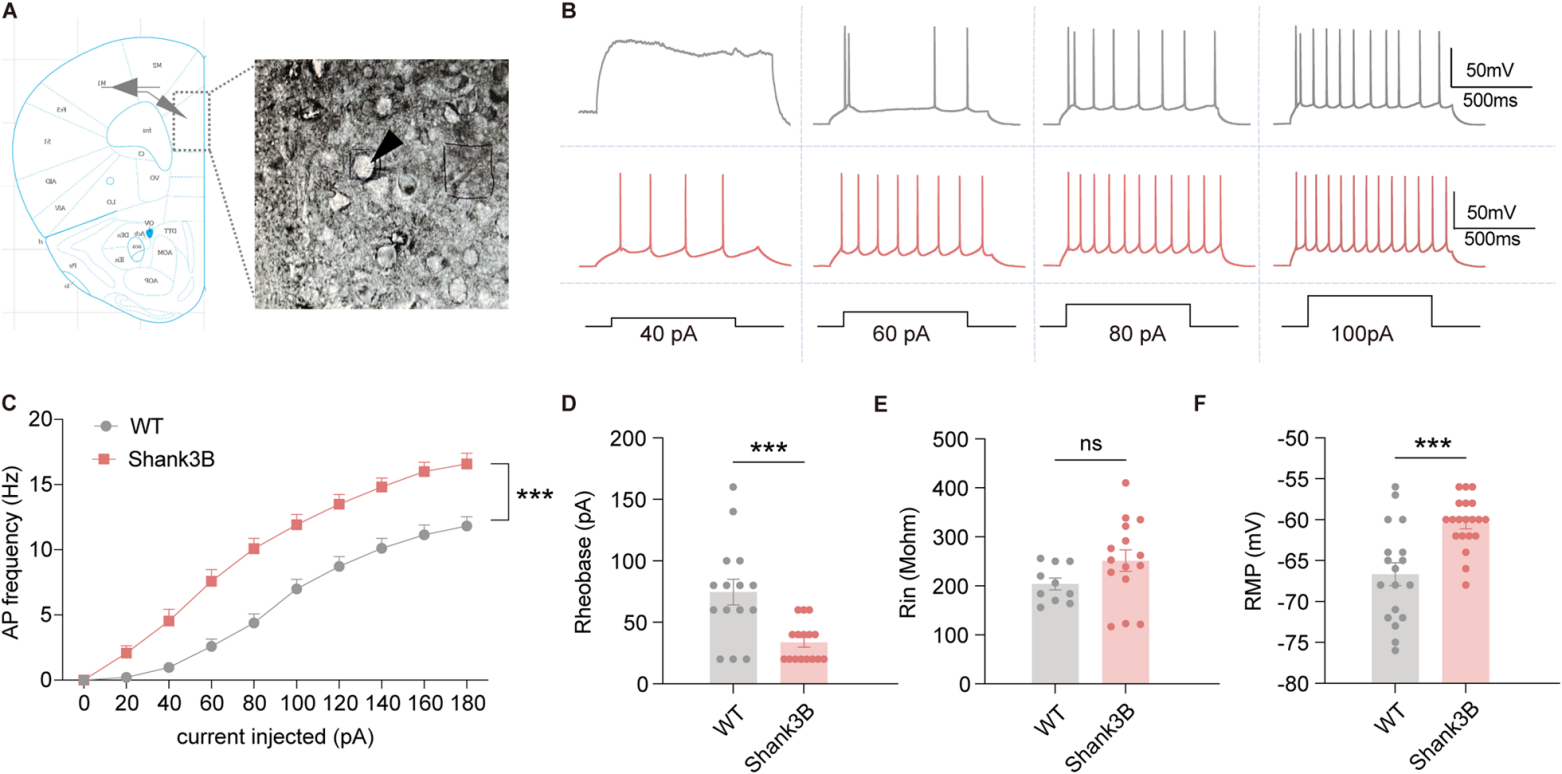
The electrophysiological properties of mPFC pyramidal neurons in Shank3B−/− mice. **A** Schematic of whole-cell patch in mPFC. **B** Representative traces of action potential (APs) in response to 500-ms-long step currents. **C** Plots of AP frequency against current injections (WT: n = 15 neurons from 5 mice; Shank3B: n = 16 neurons from 5 mice). **D** Step current injections (increment: 20 pA) crossing threshold was performed to determine neurons’ short pulse (3 ms) rheobase (WT: n = 15 neurons from 5 mice; Shank3B: n = 16 neurons from 5 mice). **E** The input resistance of mPFC PNs from WT and *Shank3B*^−/−^ mice was calculated by Ohm’s law from (WT: n = 10 neurons from 4 mice; Shank3B: n = 15 neurons from 5 mice). **F** The resting membrane potential of recorded neurons. -mV (WT: n = 18 neurons from 5 mice; Shank3B: n = 20 neurons from 6 mice). Data are presented as the mean ± sem. ***P < 0.001; ns, no significant difference

To investigate the intrinsic excitability of cortical neurons in the mPFC of *Shank3b*^−/−^ mice, we conducted whole-cell patch-clamp recordings on neurons from both WT and *Shank3b*^−/−^ mice (Fig. 1A). Current-clamp recordings revealed that *Shank3b*^−/−^ mice exhibited a marked increase in AP firing frequency in response to depolarizing current steps (0–200 pA) compared with WT controls (Fig. 1B, C). Notably, the rheobase (the minimum current required to elicit APs) was significantly lower in *Shank3b*^−/−^ mice (Fig. 1D), indicating a reduced excitability threshold. Additionally, Input resistance (Fig. 1E) showed no significant difference between the two groups, whereas the resting membrane potential (Fig. 1F) was more depolarized in Shank3b−/− mice than in WT mice.

**Fig. 2:**
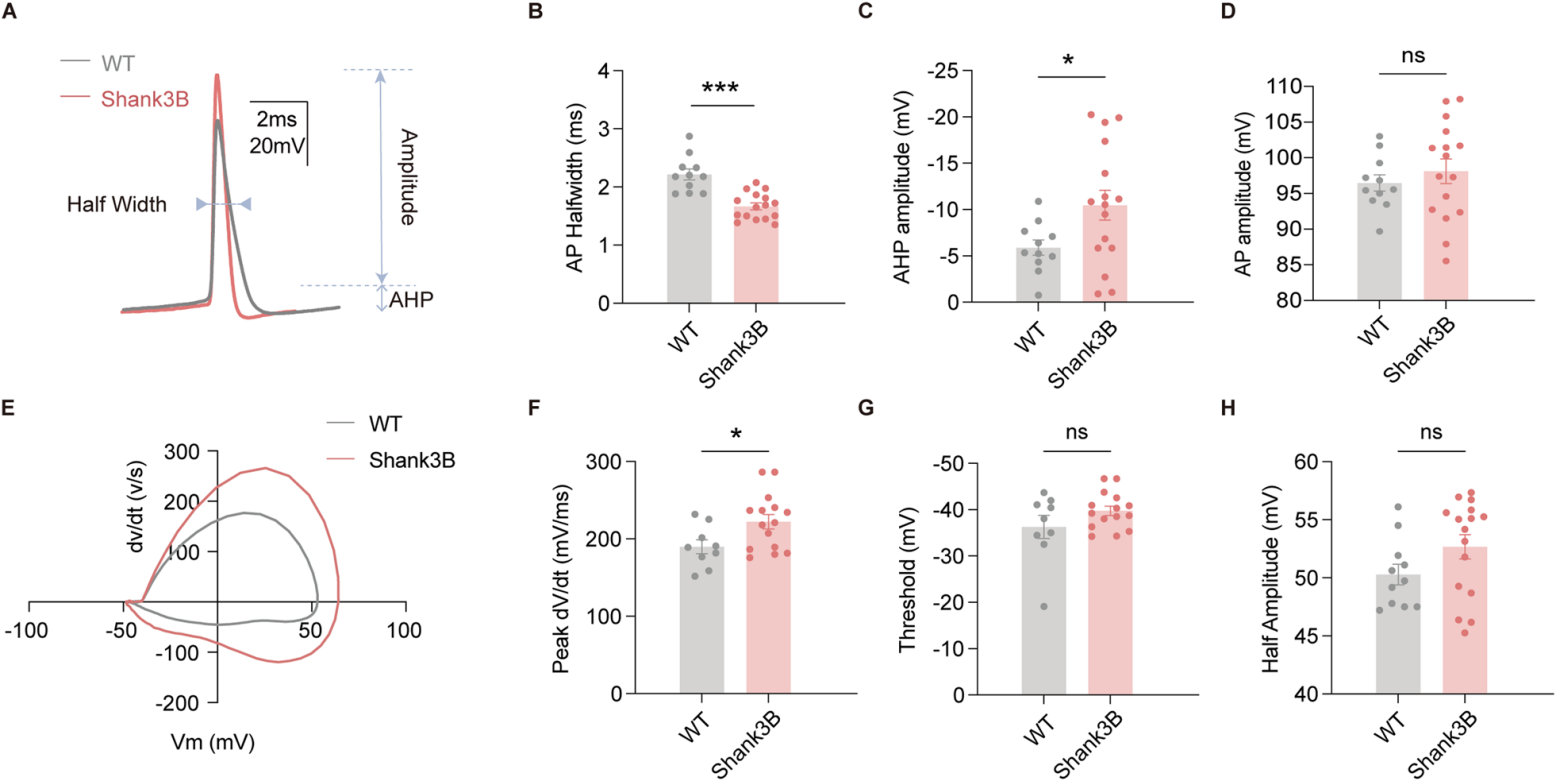
mPFC pyramidal neurons were hyperactivated in Shank3B−/− mice. **A** Representative image showing the half-width of mPFC pyramidal neurons in WT (gray trace) and Shank3B−/− mice (pink trace). **B**–**D**, **H** Quantification of AP half-width, fast after-hyperpolarization (AHP) amplitudes, AP Amplitude and AP half-amplitude (WT: n = 11 neurons from 4 mice; Shank3B: n = 16 neurons from 5 mice). **E** Phase-plane plot of the first AP at rheobase for individual neurons in the WT and Shank3B^−/−^ groups. **F**, **G** Quantification of peak rising phase dV/dt and AP threshold (WT: n = 9 neurons from 4 mice; Shank3B: n = 15 neurons from 5 mice). Data are presented as the mean ± sem. *P < 0.05; ***P < 0.001; ns, no significant difference

Regarding the intrinsic electrophysiological properties, an analysis of the AP characteristics demonstrated that *Shank3b*^−/−^ neurons fired narrower APs than WT neurons (Fig. 2A, B). Additionally, the fast AHP amplitude was significantly larger in *Shank3b*^−/−^ neurons, whereas the AP amplitude remained unchanged (Fig. 2C, D). A phase-space analysis of individual APs revealed that *Shank3b*^−/−^ neurons exhibited steeper rising phases (Fig. 2E), evidenced by a higher peak dv/dt (i.e., the maximum rate of membrane depolarization), compared with WT neurons (Fig. 2F). Notably, the threshold-crossing dynamics (i.e., the time to threshold from resting potential) were unaltered, which indicates preserved temporal precision in AP initiation (Fig. 2G). Together, these findings indicate the presence of significant alterations in membrane properties and excitability in the mPFC of *Shank3b*^−/−^ mice.

### Reduced sIPSC frequency of mPFC PNs in ***Shank3b***^***−/−***^ mice

**Fig. 3:**
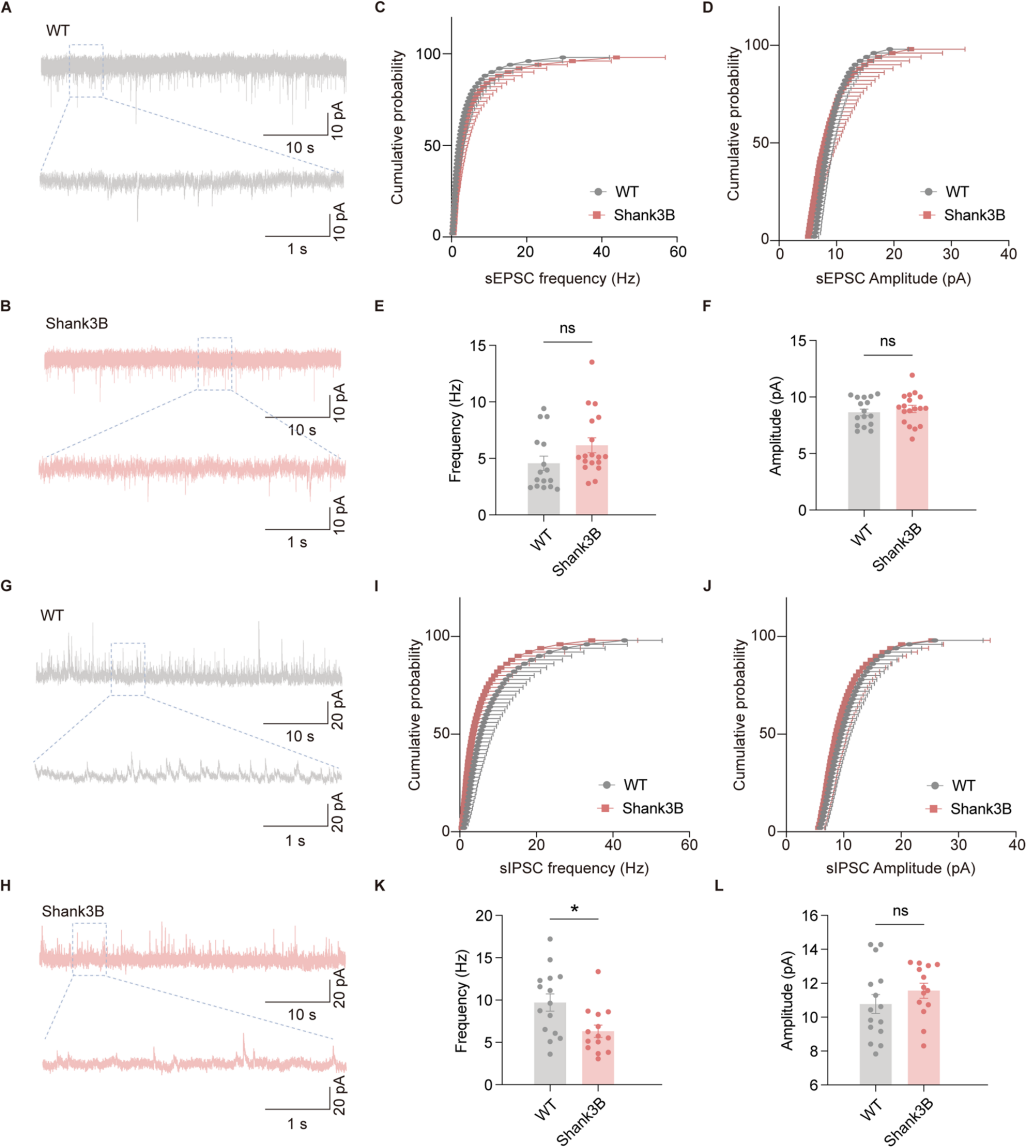
Abnormal sIPSC frequency of mPFC pyramidal neurons of Shank3B−/− mice. **A**, **B** Representative traces of sEPSCs in PNs of WT and Shank3B−/− mice. **C**, **D** Cumulative distribution of sEPSC frequency and amplitude. **E**, **F** Quantification of the data in A and B, the frequency (E) and amplitude (F) of sEPSCs.(WT: n=16 neurons from 5 mice; Shank3B: n=18 neurons from mice.) **G**, **H** Representative traces of sIPSCs in PNs of WT and Shank3B^−/−^ mice. **I**, **J** Cumulative distribution of sIPSC frequency and amplitude. **K**, **L** Quantification of the data in G and H, the frequency (K) and amplitude (L) of sIPSCs. (WT: n = 15 neurons from 5 mice; Shank3B: n = 14 neurons from 5 mice). Data are presented as the mean ± sem. *P < 0.05; ns, no significant difference

To further characterize the synaptic inputs onto mPFC PNs in *Shank3b*^*−/−*^ mice, we analyzed spontaneous synaptic currents using voltage-clamp recordings. Initially, to isolate sEPSCs, recordings were performed on PNs at a holding potential of − 70 mV in the presence of GABA receptor antagonists (to block inhibitory inputs). This analysis revealed no significant differences in either the frequency or amplitude of sEPSCs between WT and *Shank3b*^*−/−*^ mice (Fig. 3A–F), indicating that excitatory synaptic inputs onto PNs remain unaffected in the absence of *Shank3b*. Subsequently, to investigate sIPSCs, a cesium-based internal solution was used to block potassium currents and isolate inhibitory events, and recordings were conducted at 0 mV, which is a potential that favors inward chloride (Cl⁻) currents through GABA_A_ receptors. Strikingly, *Shank3b*^*−/−*^ mice demonstrated a significant reduction in sIPSC frequency compared with WT mice, whereas the sIPSC amplitude remained unchanged (Fig. 3G–L). This reduction in sIPSC frequency in mPFC PNs indicates a decline in spontaneous inhibitory synaptic transmission.

### Altered excitability and inhibitory synaptic transmission of mPFC PNs in ***Shank3b***^***−/−***^ mice

**Fig. 4:**
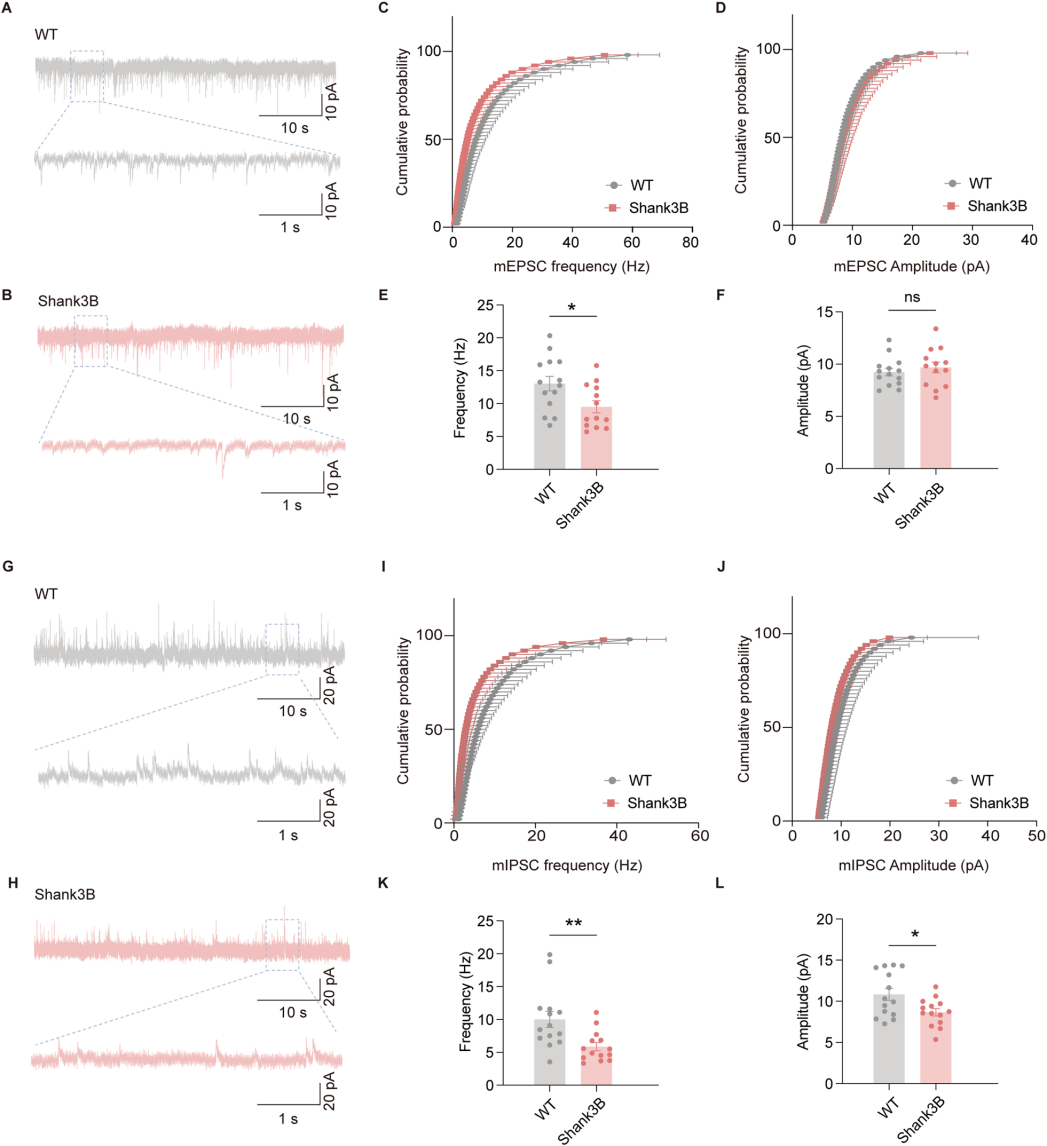
Abnormal mIPSC frequency and amplitude of mPFC pyramidal neurons in Shank3B−/− mice. **A**, **B** Representative traces of mEPSCs in PNs of WT and Shank3B−/− mice. **C**, **D** Cumulative distribution of mEPSC frequency and amplitude. **E**, **F** Quantification of the data in A and B, the frequency (E) and amplitude (F) of mEPSCs (WT: n = 14 neurons from 5 mice; Shank3B: n = 13 neurons from 5 mice). **G**, **H** Representative traces of mIPSCs in PNs of WT and Shank3B^−/−^ mice. **I**, **J** Cumulative distribution of mIPSC frequency and amplitude. **K**, **L** Quantification of the data in G and H, the frequency (K) and amplitude (L) of mIPSCs (WT: n = 14 neurons from 5 mice; Shank3B: n = 14 neurons from 5 mice). Data are presented as the mean ± sem. *P < 0.05; **P < 0.01; ns, no significant difference

To elucidate whether the altered excitability and inhibitory synaptic transmission in *Shank3b*^*−/−*^ mice are attributable to individual synapses or global network changes, we conducted a detailed analysis of mEPSCs and mIPSCs in mPFC PNs. This analysis was performed with TTX (1 μM) application to inhibit APs and the use of a cesium-based internal solution to isolate synaptic events. In the presence of TTX, *Shank3b*^*−/−*^ PNs demonstrated a significant reduction in mEPSC frequency compared with WT controls (Fig. 4A–C, E), whereas mEPSC amplitude remained unchanged (Fig. 4D, F). For inhibitory synapses, *Shank3b*^*−/−*^ PNs showed marked reductions in both mIPSC frequency and amplitude (Fig. 4G–L). This dual impairment points to disrupted GABA release machinery or altered GABA_A_ receptor trafficking, which is consistent with the reduced inhibitory drive that was observed in sIPSC recordings. We propose the following potential mechanism: reduced inhibitory input may trigger compensatory synaptic plasticity (i.e., long-term potentiation at glutamatergic synapses), thus leading to the passive enhancement of excitatory signaling to maintain neuronal activity.

### Morphological changes of mPFC PNs in ***Shank3b***^***−/−***^ mice

**Fig. 5:**
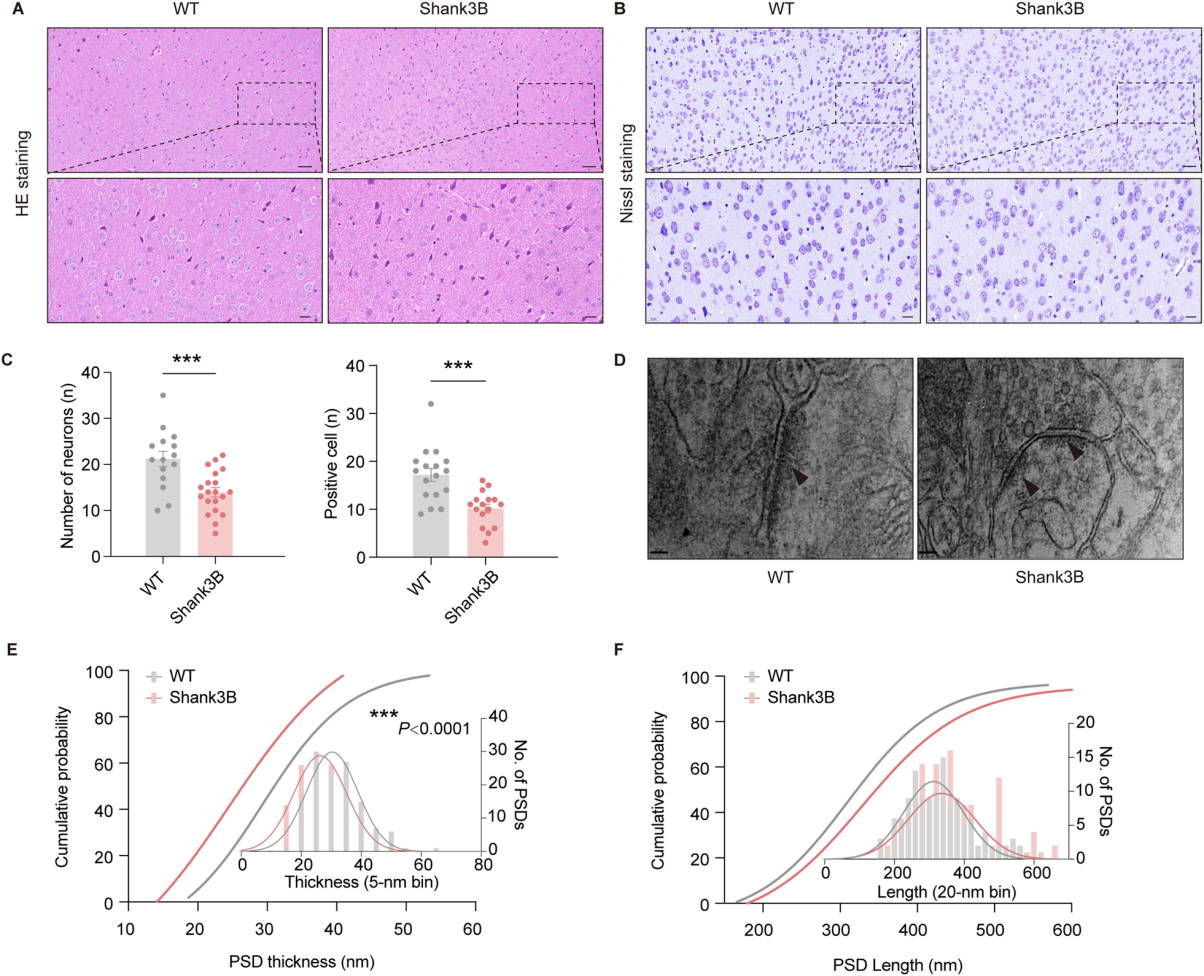
Morphological changes of mPFC pyramidal neurons in Shank3B−/− mice **A**–**C** H&E and Nissl images from the ipsilateral side of the mPFC. n = 3 mice per group. H&E showed swollen cells with deeply stained cytoplasm, and Nissl showed loss of Nissl vesicles and nuclear fragmentation. **D**–**F** Examples of electron micrographs depicting the synaptic contacts with presynaptic vesicles, postsynaptic densities (arrow); scale bar, 20 nm. ***P < 0.001

We subsequently conducted pathological analyses of ***Shank3b***^*−/−*^ mice, which revealed disrupted cellular organization and a significant reduction in neuronal count, as evidenced by hematoxylin and eosin staining (Fig. 5A, C). Additionally, Nissl staining results indicated a marked decrease in the number of Nissl-positive cells and a loss of Nissl vesicles in ***Shank3b***^*−/−*^ mice compared with WT mice (Fig. 5B, D).

To investigate alterations in postsynaptic morphology, we used TEM to examine PSD structures. We observed a significant reduction in the mean thickness of PSDs in ***Shank3b***^*−/−*^ mice compared with WT mice (Fig. 5E). However, no significant difference in PSD length was detected between the ***Shank3b***^*−/−*^ and WT mice (Fig. 5F). This absence of variation in PSD length despite the previously reported evidence of synaptic dysfunction suggests that *Shank3b* specifically influences the structural organization of the PSD rather than its overall morphological scaling.

### RNA-seq reveals divergent gene expression profiles and enriched pathways in *Shank3b*-deficient mice

**Fig. 6:**
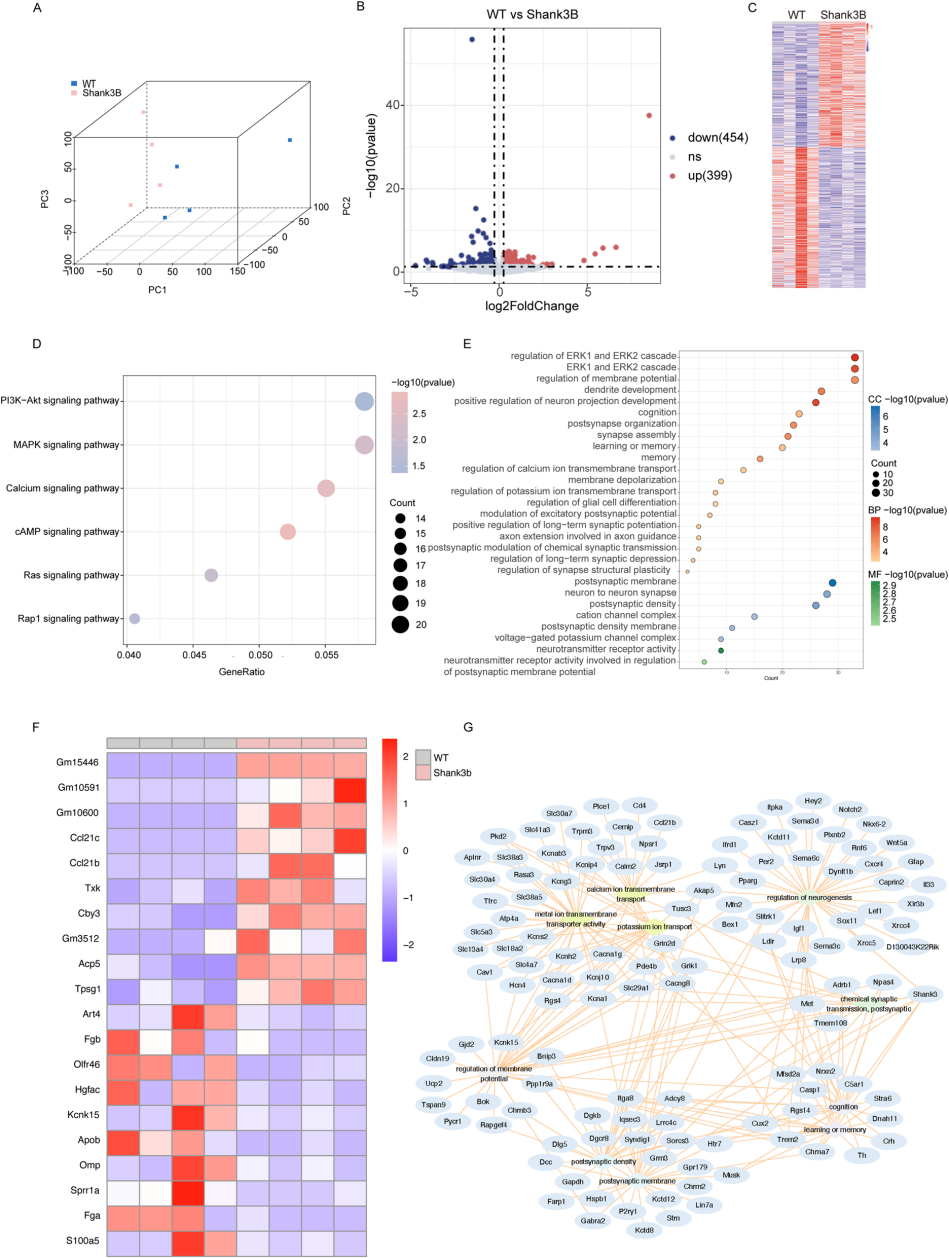
RNA Sequencing Reveals Divergent Gene Expression Profiles and Enriched Pathways in Shank3B-Deficient Mice. **A** Principal component analysis (PCA) among the two sample groups. **B** Volcano plot of differentially expressed genes (DEGs). **C** Heat map of significant differential gene expression among the two sample groups. **D** KEGG enrichment of the differential genes between the two sample groups. **E** GO enrichment of the differential genes between the two sample groups. **F** Heatmap of selected DEGs. **G** Protein–protein interaction (PPI) network of hub DEGs. (WT and Shank3B: n = 4)

To identify the transcriptomic alterations underlying *Shank3b* deficiency, we performed RNA-seq on PFC tissue from WT and *Shank3b*^*−/−*^ mice (n = 4 per group). Hierarchical clustering confirmed distinct transcriptional separation between the two genotypes (Fig. 6A), thus supporting the robustness of the dataset. Differential expression analysis using DESeq2 (v4.3.2) with thresholds of |log_2_ fold-change|> 1 and false discovery rate < 0.05 identified 853 DEGs in *Shank3b*^*−/−*^ vs. WT mice, including 399 upregulated and 454 downregulated transcripts (Fig. 6B, C).

GO enrichment analysis revealed that *Shank3b*^*−/−*^ mice exhibited a significantly higher expression of DEGs in biological processes related to synaptic function, neuronal development, and signal transduction compared with WT mice. The enriched pathways primarily encompassed processes such as “dendrite development,” “positive regulation of neuron projection development,” “postsynapse organization,” “synapse assembly,” and “regulation of synapse structural plasticity” (Fig. 6D). Additionally, KEGG pathway analysis highlighted pathways that are associated with neurodevelopmental and synaptic signaling. The most significantly enriched pathway was the “PI3K-Akt signaling pathway;” this includes genes such as *Igf1*, *Fgfr1*, and *Chrm2*, which are all critical for synaptic plasticity. Additional enriched pathways included “axon guidance,” “Ras signaling pathway,” and “MAPK signaling pathway,” thereby suggesting disrupted axonal growth and intracellular signaling cascades in *Shank3b*^*−/−*^ mice (Fig. 6E). The most specific pathway among these was the “calcium signaling pathway,” which includes genes such as *Itpka* and *Grin2d*. This finding suggests that the phenotype of *Shank3b*^*−/−*^ mice is linked to calcium ion channels, which is consistent with the results of our patch-clamp electrophysiological recordings.

The heatmap of the top 20 genes visualizes the expression profiles across the two groups (Fig. 6F). A network analysis of differential gene expression in the mPFC between *Shank3b*^*−/−*^ and WT mice revealed that the DEGs formed a complex interaction network with key functional terms. Modules that are associated with biological processes—including calcium ion transport, transmembrane transporter activity, neurogenesis regulation, postsynaptic chemical signal transmission, and cognitive function—were significantly enriched. Moreover, the nodes within these modules (genes/functional terms) exhibited strong interactions via dense connections, which indicates that *Shank3b* deficiency may disrupt ion homeostasis, synaptic transmission, and neurodevelopment-related pathways, thereby driving dysfunction in the mPFC (Fig. 6G). Collectively, these results suggest that *Shank3b* deficiency disrupts genes and pathways that are central to synaptic structure and neural development, thus providing a molecular framework for understanding its phenotypic consequences.

## Discussion

ASD is a neurodevelopmental condition that is characterized by social communication deficits, repetitive behaviors, and cognitive impairments; synaptic dysfunction and E/I imbalance have been proposed as core pathophysiological mechanisms [1]. The *SHANK3B* gene, which encodes a key PSD scaffolding protein, is a high-risk genetic factor for ASD, and its deficiency disrupts synaptic structure and function across multiple brain regions [5, 9, 10]. However, the region-specific mechanisms by which *Shank3b* deficiency impairs mPFC circuitry—which is critical for social cognition and emotional regulation [17, 18]—remain incompletely understood. In the present study, we integrated behavioral, electrophysiological, morphological, and transcriptomic analyses in *Shank3b*^−/−^ mice to elucidate the effects of *Shank3b* deficiency on mPFC PNs. Our findings revealed enhanced intrinsic excitability, impaired GABAergic synaptic transmission, and dysregulated synaptic gene expression, thus providing new insights into the mPFC-specific pathophysiology of *SHANK3B*-related ASD.

### *Shank3b* deficiency induces mPFC PN hyperexcitability, aligning with ASD-related hyperexcitability phenotypes

A central finding of the present study was the increased intrinsic excitability of mPFC layer V PNs in *Shank3b*^*−/−*^ mice, as evidenced by higher AP firing frequency, lower rheobase, and depolarized resting membrane potential (Fig. 1).

This finding contrasts with some prior *Shank3* models that reportedly have reduced intrinsic excitability. For example, *Shank3Δ11*^−/−^ mice are reported to exhibit hypoexcitability in striatal medium spiny neurons because of downregulated K_v_1 potassium channels. However, our findings are largely consistent with the results reported in the majority of studies. For example, striatal medium spiny neurons and hippocampal CA1 PNs also exhibit reduced AP thresholds and increased firing rates [21, 22]. In addition, a study by Devienne et al. noted early synaptic deficits at postnatal day 14 within the mPFC network activity and the reduced excitability of excitatory neurons, which suggests overall hypofunction [23]. By adulthood, these mice were characterized by mPFC network hyperfunction and layer V pyramidal cell hyperexcitability [23]. We have expanded this description of enhanced neuronal excitability to include more details.

Our findings align with the emerging evidence of compensatory ion channel remodeling in *Shank3* deficiency. The observed narrowing of AP half-width and enhanced fast AHP amplitude (Fig. 2) suggest the existence of altered Na^+^/K^+^ channel dynamics, and potentially reflect homeostatic adjustments to maintain AP fidelity amid synaptic dysfunction [24, 25]. Phase-space analysis further revealed steeper AP rising phases (higher peak dV/dt) without threshold-crossing changes (Fig. 2E–G), thus indicating accelerated Na⁺ channel activation kinetics; this is consistent with studies in ASD models showing altered sodium channel protein (SCN)1A/SCN2A sodium channel function [26].

Importantly, this intrinsic hyperexcitability likely contributes to sensory hypersensitivity and anxiety-like behaviors in *Shank3b*^*−/−*^ mice, which mirrors clinical reports of heightened sensory reactivity in individuals with ASD.

### *Shank3b* deficiency selectively impairs GABAergic synaptic transmission in the mPFC, disrupting the E/I balance

The E/I balance is a cornerstone of neural circuit homeostasis, and its disruption is widely recognized as a central mechanism in ASD pathogenesis. Our study revealed a complex E/I imbalance in mPFC PNs of *Shank3b*^−/−^ mice; there was a selective impairment of inhibitory synaptic transmission (i.e., a reduced frequency/amplitude of sIPSCs and mIPSCs; Figs. 3, 4), alongside a modest reduction in excitatory transmission (i.e., a significant decrease in mEPSC frequency but preserved mEPSC amplitude; Fig. 4A–F). This region-specific imbalance, which was driven by combined hypoinhibition and partial excitatory hypoactivity, aligns with the critical role of the mPFC in the top-down regulation of social/emotional behavior and its established link to ASD-related social deficits.

Another finding of our study was a modest reduction in mEPSC frequency (with preserved amplitude) in *Shank3b*^−/−^ mPFC neurons, which contrasts with previous reports of severe excitatory deficits in other *Shank3* models and brain regions. For example, Jiang et al. reported reduced NMDA receptor currents and corticostriatal excitation in *Shank3* mutant mice, which was driven by impaired activity-dependent synaptic scaling [5]. This discrepancy likely reflects regional heterogeneity in synaptic plasticity and compensatory mechanisms. The mPFC, which is specialized for top-down cognitive control, may use unique strategies to stabilize excitatory networks.

Our observation of reduced IPSC frequency/amplitude in *Shank3b*^−/−^ mPFC neurons reinforces the critical role of *Shank3* in maintaining inhibitory synapse integrity, which is consistent with prior reports in other brain regions. SHANK3 is a postsynaptic scaffolding protein that organizes glutamate receptors and associated signaling molecules at excitatory synapses but also interacts with GABAergic receptors, which makes it essential for inhibitory synapse stability. For example, Jurkovičová-Tarabová et al. reported decreased mIPSC frequency in the hippocampal PNs of *Shank3b*^−/−^ mice, which they attributed to reduced presynaptic GABA release probability caused by disrupted vesicle docking [27]. Similarly, Bačová et al. reported that *Shank3* deficiency leads to alterations in GABAergic neurons and impaired GABAergic function in dopaminergic brain areas of *Shank3*^−/−^ mice, which may then cause hyperactivity and repetitive behaviors [28]. These studies collectively indicate that *Shank3* deficiency compromises GABAergic transmission across circuits, and our work extends this to the mPFC—a region that is critical for social cognition but understudied in *Shank3* models.

Notably, recent work has linked GABA_A_ receptor subunit abnormalities in the mPFC to ASD-like social deficits in rodents [29]. Our data align with this idea because reduced mIPSC frequency and amplitude likely reflect both presynaptic GABA release defects and postsynaptic GABA_A_ receptor dysfunction in mPFC pyramidal interneurons.

The role of the mPFC in integrating sensory and emotional signals for social decision-making means that its E/I imbalance is particularly relevant to ASD. Our data indicate that *Shank3b* deficiency impairs mPFC inhibition (via GABA-mediated IPSCs) and partially dampens excitation, thus tipping the balance toward functional hypoactivity during social tasks. This aligns with the report that low-frequency repetitive transcranial magnetic stimulation (an inhibitory protocol) can ameliorate ASD-like behaviors in rats by restoring the E/I imbalance through enhancing local cortical inhibition [30]. Moreover, the optogenetic activation of mPFC parvalbumin interneurons in *Shank3b*^−/−^ mice rescues social interaction deficits [31], thereby directly implicating local GABA-mediated inhibition in ASD pathology.

### Synaptic and morphological deficits in the ***Shank3b***^*−/−*^ mPFC: linking structure to function

Our morphological analyses revealed that *Shank3b* deficiency caused significant neuronal loss, reduced Nissl body density, and decreased PSD thickness in mPFC PNs (Fig. 5) [11]. The preservation of PSD length despite a reduced PSD thickness (Fig. 5E–F) indicates that *Shank3b* may specifically regulate PSD structural organization rather than overall synaptic scaling, which is consistent with its role as a PSD scaffolding protein [9, 32]. These structural anomalies likely contributed to the observed functional deficits in synaptic transmission; reduced PSD thickness may impair receptor clustering (GABA_A_ receptors), whereas neuronal loss reduces the number of functional synapses [4, 8].

Notably, the mPFC of *Shank3b*^*−/−*^ mice has also been reported to exhibit reduced dendritic branching and dysregulated NMDA/AMPA receptor ratios in prior studies [20, 21]. Together with our findings, this suggests that *Shank3b* deficiency disrupts both synaptic structure and function in a region-specific manner.

### Transcriptomic dysregulation: synaptic plasticity and calcium signaling pathways in *Shank3b* deficiency

Our RNA-seq analysis identified 853 DEGs in the *Shank3b*^*−/−*^ mPFC, which were enriched in pathways related to synaptic function, calcium signaling, and neurodevelopment (Fig. 6) [15]. GO enrichment revealed the upregulation of genes involved in “postsynapse organization” and “synapse assembly” (Fig. 6D), in contrast to the observed PSD thinning, which suggests a compensatory but ineffective attempt to maintain synaptic integrity [10]. KEGG analysis highlighted the calcium signaling pathway (*Itpka*, *Grin2d*) as a key dysregulated pathway (Fig. 6E), thereby implicating calcium-dependent processes in *Shank3b*^*−/−*^ pathophysiology [33, 34]. This result aligns with our electrophysiological data that showed altered AP dynamics, because calcium channels regulate AP shape and synaptic plasticity [35, 36].

The phosphatidylinositol 3-kinase (PI3K)/protein kinase B (Akt) signaling pathway, which is critical for synaptic plasticity, was also enriched in the present study (Fig. 6E) [9]. Genes in this pathway, such as *Igf1* and *Fgfr1*, can modulate dendritic growth and synapse formation [37, 38]. and their dysregulation may contribute to the reduced PSD thickness and neuronal loss that were observed in the *Shank3b*^*−/−*^ mPFC.

### Implications for ASD pathogenesis and therapeutic strategies

Our study positions *Shank3b* as a critical regulator of mPFC circuit homeostasis, with its deficiency driving ASD-like phenotypes through E/I imbalance, synaptic structural defects, and dysregulated gene expression. The role of the mPFC in social cognition and emotional regulation [17, 18] makes it a key target for intervention. Therapeutically, targeting GABAergic signaling (via GABA_A_ receptor modulators) or normalizing intrinsic excitability (via sodium channel inhibitors) might restore the mPFC E/I balance [20, 22]. Our RNA-seq data further identified the calcium signaling and PI3K/Akt pathways as potential targets for future pharmacological or gene therapy approaches [9, 33].

### Limitations and future directions

The present study had several limitations. First, we focused on 6- to 8-week-old mice only, and age-dependent changes in *Shank3b*-related phenotypes remain unexplored [20]. Second, although we identified presynaptic and postsynaptic deficits in GABAergic transmission, the specific molecular players (i.e., GABA_A_ receptor subunits) require further investigation [14]. Third, it will be essential to integrate optogenetic techniques with behavioral assays to assess the contribution of GABAergic impairment in the PFC to behavioral abnormalities. Finally, sex differences in *Shank3b*-related phenotypes were not addressed, despite evidence of sex-specific differences in ASD prevalence [2, 9].

## Conclusions

Our findings indicate that *Shank3b* deficiency disrupts mPFC circuitry by enhancing intrinsic excitability, impairing GABAergic synaptic transmission, and dysregulating synaptic gene expression. These alterations led to deficits in synaptic and neuronal number and morphology as well as changes in the expression of synapse-associated genes. Together, these findings highlight the mPFC as a critical region for *SHANK3B*-related ASD pathology and provide a framework for developing circuit-based therapies that target the E/I balance and synaptic function.

## Supplementary Information

**Additional file 1**: Supplement 1. Behavioral characterization of wild-type (WT) and Shank3B mice in the open-field test and three-chamber social test. (A) Representative locomotor trajectories of WT (upper panel) and Shank3B (lower panel) mice in the open-field arena. (B–G) Behavioral metrics in the open-field test: (B) Number of entries into the central zone; (C) Percentage of time spent in the central zone; (D) Distance traveled within the central zone; (E) Number of rearings; (F) Total distance traveled across the entire arena; (G) Duration of self-grooming behavior. (H) Heatmap visualization of exploratory activity and spatial distribution during the sociality phase (S1: compartment with a novel social stimulus; EM: compartment with an empty module). (I) Percentage of time spent in compartments containing the social stimulus (S1) vs. the empty module (EM). (J) Total sniffing duration directed toward the social stimulus (S1) and the empty module (EM). (K) Preference index for the social stimulus over the empty module. (L) Heatmap visualization of exploratory activity and spatial distribution during the social novelty phase (S1: compartment with a familiar mouse; S2: compartment with a novel mouse). (M) Percentage of time spent in compartments containing the familiar (S1) vs. novel (S2) mouse. (N) Total sniffing duration directed toward the familiar (S1) and novel (S2) mice. (O) Preference index for the novel mouse over the familiar mouse. Data are presented as mean ± standard error of the mean (SEM). Statistical significance was determined by an unpaired two-tailed Student’s t-test. **P < 0.01, *P < 0.05, “ns” indicates no significant difference

## Data Availability

No datasets were generated or analysed during the current study.
