# Supplementary figures and images for: Shank3B deficiency disrupts GABAergic synaptic transmission in pyramidal neurons of the medial prefrontal cortex region in autism spectrum disorder
Source: Mol Brain. 2026 Mar 9;19:23. doi: 10.1186/s13041-026-01289-z (PMC13069784; doi:10.1186/s13041-026-01289-z)

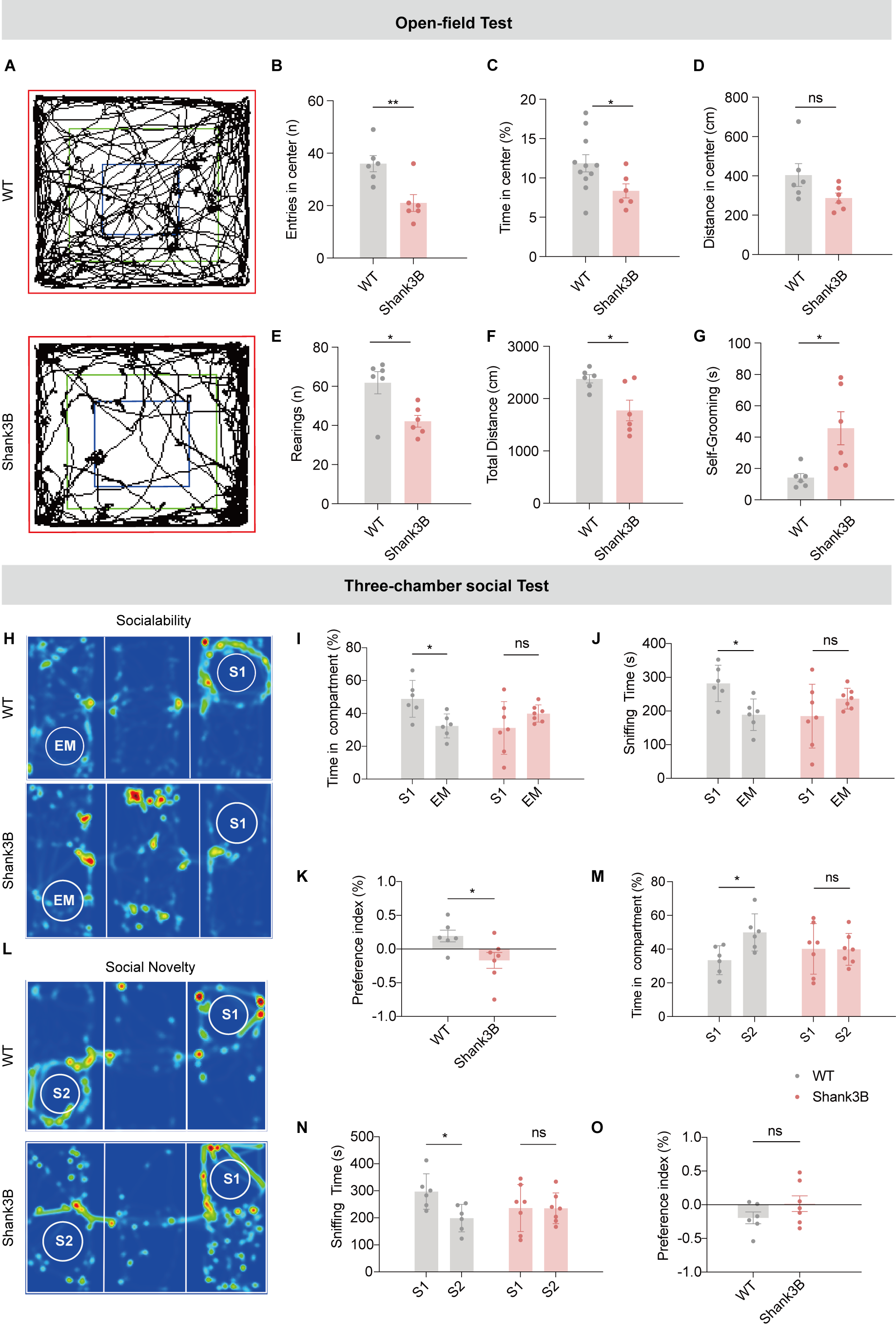

Supplement: Supplementary file 1 — Additional file 1: Supplement 1. Behavioral characterization of wild-type (WT) and Shank3B mice in the open-field test and three-chamber social test. (A) Representative locomotor trajectories of WT (upper panel) and Shank3B (lower panel) mice in the open-field arena. (B–G) Behavioral metrics in the open-field test: (B) Number of entries into the central zone; (C) Percentage of time spent in the central zone; (D) Distance traveled within the central zone; (E) Number of rearings; (F) Total distance traveled across the entire arena; (G) Duration of self-grooming behavior. (H) Heatmap visualization of exploratory activity and spatial distribution during the sociality phase (S1: compartment with a novel social stimulus; EM: compartment with an empty module). (I) Percentage of time spent in compartments containing the social stimulus (S1) vs. the empty module (EM). (J) Total sniffing duration directed toward the social stimulus (S1) and the empty module (EM). (K) Preference index for the social stimulus over the empty module. (L) Heatmap visualization of exploratory activity and spatial distribution during the social novelty phase (S1: compartment with a familiar mouse; S2: compartment with a novel mouse). (M) Percentage of time spent in compartments containing the familiar (S1) vs. novel (S2) mouse. (N) Total sniffing duration directed toward the familiar (S1) and novel (S2) mice. (O) Preference index for the novel mouse over the familiar mouse. Data are presented as mean ± standard error of the mean (SEM). Statistical significance was determined by an unpaired two-tailed Student’s t-test. **P < 0.01, *P < 0.05, “ns” indicates no significant difference [file 13041_2026_1289_MOESM1_ESM.tif]
